# MicroRNA-224 is Readily Detectable in Urine of Individuals with Diabetes Mellitus and is a Potential Indicator of Beta-Cell Demise

**DOI:** 10.3390/genes6020399

**Published:** 2015-06-23

**Authors:** Siobhán Bacon, Britta Engelbrecht, Jasmin Schmid, Shona Pfeiffer, Ross Gallagher, Ailbhe McCarthy, Marie Burke, Caoimhín Concannon, Jochen H. M. Prehn, Maria M. Byrne

**Affiliations:** 1 Department of Endocrinology, Mater Misericordiae University Hospital, Eccles Street, Dublin 7, Ireland; E-Mails: siobhanbacon@gmail.com (S.B.); ailbhe.mccarthy@gmail.com (A.M.); mburke@mater.ie (M.B.); mbyrne@mater.ie (M.M.B.); 2 Departments of Physiology and Medical Physics, Royal College of Surgeons in Ireland, 123 St Stephen’s Green, Dublin 2, Ireland; E-Mails: brittaengelbrecht@gmx.de (B.E.); jasmin.schmid@gmail.com (J.S.); shonapfeiffer@rcsi.ie (S.P.); rossgallagher@rcsi.ie (R.G.); cconcannon@rcsi.ie (C.C.); 3 Centre for Systems Medicine, Royal College of Surgeons in Ireland, 123 St Stephen’s Green, Dublin 2, Ireland

**Keywords:** diabetes associated microRNA, miR-103, miR-224, urinary biomarker, insulin secretion

## Abstract

MicroRNA (miRNA) are a class of non-coding, 19–25 nucleotide RNA critical for network-level regulation of gene expression. miRNA serve as paracrine signaling molecules. Using an unbiased array approach, we previously identified elevated levels of miR-224 and miR-103 to be associated with a monogenic form of diabetes; HNF1A-MODY. miR-224 is a novel miRNA in the field of diabetes. We sought to explore the role of miR-224 as a potential biomarker in diabetes, and whether such diabetes-associated-miRNA can also be detected in the urine of patients. Absolute levels of miR-224 and miR-103 were determined in the urine of *n* = 144 individuals including carriers of a HNF1A mutation, participants with type 1 diabetes mellitus (T1DM), type 2 diabetes mellitus (T2DM) and normal controls. Expression levels were correlated with clinical and biochemical parameters. miR-224 was significantly elevated in the urine of carriers of a HNF1A mutation and participants with T1DM. miR-103 was highly expressed in urine across all diabetes cohorts when compared to controls. For both miR-224 and-103, we found a significant correlation between serum and urine levels (*p* < 0.01). We demonstrate that miRNA can be readily detected in the urine independent of clinical indices of renal dysfunction. We surmise that the differential expression levels of miR-224 in both HNF1A-MODY mutation carriers and T1DM may be an attempt to compensate for beta-cell demise.

## 1. Introduction

miRNA have emerged as potent regulators of glucose homeostasis. miRNA are 19–25 nucleotide non-coding RNA molecules which suppress gene expression via imperfect base pairing to the 3' untranslated region of target mRNAs, leading to repression of protein production or mRNA degradation. miRNA have been shown to play a crucial role in pancreatic development [1,2], insulin secretion [2,3,4] and insulin resistance [5]. In addition to being potential therapeutic targets, miRNA are also considered as future diagnostics and biomarkers for disease progression and therapeutic responses. They are differentially expressed in tissues affected by diabetes when compared to normal controls, and have been detected in serum, plasma and whole blood in animal models and human subjects with diabetes [6,7,8]. Unique miRNA profiles have been identified in the serum of participants with diabetes, including both paediatric and adult cohorts [9,10,11,12,13,14,15]. miRNA have also been identified as biomarkers for micro- and macrovascular complications with the potential to be of clinical utility in diabetes care [16,17,18,19]. The role of circulating miRNA in diabetes has been studied almost exclusively using the serum of human subjects. However, microRNA detection in various more accessible body fluids has attracted recent attention [20].

HNF1A-maturity-onset diabetes of the young (HNF1A-MODY) is the most common monogenic form of diabetes resulting from mutations in the gene encoding the pancreatic transcription factor hepatocyte nuclear factor 1α (HNF1A) [21]. We recently demonstrated that induced suppression of endogenous HNF1A function in INS-1 cells, a cellular model of HNF1A-MODY [22,23,24], increased the levels of two specific miRNA; miR-224 and miR-103 [25]. Using absolute quantitative PCR analysis, we demonstrated that miR-224 and miR-103 levels were significantly elevated in the serum of HNF1A-MODY mutation carriers when compared to controls [25]. miR-103 has been previously shown to play a key role in insulin resistance and glucose homeostasis. The overexpression of miR-103 had been shown to result in impaired insulin sensitivity [5].

miRNA can be secreted from cells and accumulate in microvesicles, either bound to Ago2 protein, or as free miRNA in extracellular fluids [26,27,28]. Of note, HNF1A is expressed not only in the pancreas but also the liver, digestive tract and in particular the kidney [29]. Urine has proven to be a stable biofluid [30] which can be acquired non-invasively and is not subject to collection difficulties such as the haemolysis associated with blood draw. The aim of this current study was to obtain proof-of-concept that miR-224 and miR-103 are detectable in the urine of HNF1A-MODY mutation carriers, and to determine whether these diabetes-associated miRNA are also elevated in the urine of patients with T1DM and T2DM. We also sought to correlate urinary levels of miRNA with serum levels, and with clinically relevant indices of renal disease.

## 2. Experimental Section

### 2.1. Patient Cohorts

Individuals with a clinical diagnosis of MODY were recruited from the MODY diabetes clinic in the Mater Misericordiae University Hospital Dublin in Ireland. MODY is a monogenic, autosomal dominant form of diabetes. In contrast to type 1 diabetes mellitus (T1DM), patients with MODY are usually auto-antibody negative [islet cell antibody (ICA)/anti-glutamic acid decarboxylase (GAD)], non-ketotic and have detectable C-peptide suggesting preserved beta-cell function. Typically, at least one family member in a MODY pedigree is diagnosed under the age of 25 years. In the current study, genetically confirmed MODY carriers included 38 cases with *HNF1A* mutations. The mutations included G207D, P291finsC, S352fsdelG, F426X, P379T, E230fsdelGA, p.L502fs, p.Leu17His, p.R159Q, R200Q, S335X and V351fsdelG. Sequencing of the HNF1A gene was performed by IntegraGen (Bonn, Germany) in 2006–2007 and the Molecular Genetics Laboratory (Exeter, UK) in 2008–2014. Analysis of the HNF1A gene was performed by polymerase chain reaction (PCR) amplification of highly purified genomic DNA, followed by semi-automated unidirectional DNA sequencing of all exons, including the highly conserved flanking intronic sequences of the exon-intron splice junctions (HNF1A mRNA accession number NM_000545.5).The mutations named above have been previously published and co-segregate in families with diabetes [31,32]. The cohort with T1DM was selected based on the permanent usage of insulin since diagnosis, evidence of beta-cell dysfunction such as undetectable C-peptide and positive antibody status (GAD ± ICA). The cohort with T2DM was recruited based on not meeting the clinical criteria for a MODY diagnosis, specifically the absence of a significant family history, an older age at diagnosis and not being of lean body habitus. The normal control group was selected based on the detection of normoglycaemia, normotension and an absence of co-morbidities. Individuals were excluded from the control group if taking prescribed medication for hypertension or hyperlipidaemia. The study was approved by the Research Ethics Committee at the Mater Misericordiae University Hospital Dublin and all participants gave informed written consent.

### 2.2. Phenotyping of the Cohort

All participants underwent a clinical assessment including a full medical history and physical examination. We recorded details of any prescribed medication, in particular, antihypertensive agents, anticoagulants and lipid lowering medication. A urinary sample was collected in a fasting state and collected in a specimen jar (20 mL volume). A mid-stream clean catch void was advised. Immediately after collection, a 1 mL aliquot of urine was drawn from the specimen and immediately frozen. For miRNA analysis, 100 µL of the urine specimen was utilized. The remaining proportion was analyzed for leukocytes and the albumin/creatinine ratio (ACR). The Urinary Albumin Creatinine Ratio (UACR) was calculated as the ratio between urinary albumin and creatinine. The UACR is not affected by urine concentration. Micro-albuminuria is said to be present when the UACR is 3.4–33.9 g/mol and overt albuminuria is present when the UACR is >34 g/mol. The CKD-EPI (Chronic Kidney Disease Epidemiology Collaboration, http://www.qxmd.com/calculate-online/nephrology/ckd-epi-egfr) creatinine equation was used for glomerular filtration rate (GFR) estimation. CKD was defined as an eGFR <60 mL/min/1.73 m^2^. Urinary osmolality was also measured using the Semi-micro Osmometer K-7400 (Knauer, Berlin, Germany).

### 2.3. Oral Glucose Tolerance Test (OGTT)

A 75 g OGTT was performed on participants with HNF1A-MODY and T2DM after an overnight fast with measurement of glucose, insulin and C-peptide at baseline and 30 min intervals to determine degree of glucose intolerance and insulin secretory response. The oral glucose insulin sensitivity (OGIS) was calculated as previously described using a calculation involving the weight, height, glucose and insulin levels at timed intervals during an OGTT [33].

### 2.4. Biochemical Assays

All laboratory analyses were performed with commercially available standardized methods. The plasma glucose concentration was measured using Beckman Synchron DXC800 (Beckman Instruments Inc., Brea, CA, USA). HbA_1c_ was determined using high performance liquid chromatography (Menarini HA81-10, Rome, Italy). Insulin and C-peptide were analyzed using Immulite 2000 immunoassay (Siemens Healthcare Diagnostics, Deerfield, IL, USA). Anti-GAD_65_ antibodies were analysed by ELISA (Euroimmun, Luebeck, Germany). ICA was performed by indirect immunofluorescence test by the supra-Regional Protein Reference Unit and Dept. of Immunology in Sheffield, UK.

### 2.5. RNA Isolation from Human Urine

Following centrifugation at 3000× *g* for 10 min at 4 °C, total RNA containing small RNA was extracted from 100 µL urine using the miRNeasy Serum/Plasma kit (Qiagen, Hilden, Germany). In brief, 700 µL of QIAzol reagent was added to 100 µL of urine sample and mixed well. To allow for normalization of sample-to-sample variation in RNA isolation, 25 fmol synthetic *Caenorhabditis elegans* miRNA *cel-miR-39*, (Sigma-Aldrich, Wickloe, Ireland) was added to each sample after 5 min incubation in QIAzol. Samples were mixed followed by adding 100 µL of chloroform. After mixing vigorously for 15 s, samples were centrifugated at 12,000× *g* for 15 min at 4 °C. The upper aqueous phase was carefully transferred avoiding the interphase to a new tube, and 700 µL of ethanol was added and mixed. At that point, the manufacturer’s protocol was followed, with the entire aqueous phase from each sample loaded onto a single affinity column. Total RNA was eluted by adding 16 µL of RNase-free water to the membrane of the spin column and incubating for 1 min before centrifugation at 15,000× *g* for 1 min at room temperature and was stored at −80 °C. For the direct comparison of urine and serum miRNA levels, 200 µL urine or serum were isolated and quantified as described in Bonner *et al.* [25]. The *C*_t_ values for the qPCR are contained in Supplementary Table S1.

**Table 1:** Clinical characteristics of patient groups. n.a. = not applicable, n.s. = not statistically significant, (*) = statistically significant with Bonferroni Correction.

Parameter	Normal Controls	T1DM	T2DM	HNF1A-MODY	Statistical Analysis *p* Value (Bonferroni Corrected)					
Group	0	1	2	3	0 *vs.* 1	0 *vs.* 2	0 *vs.* 3	1 *vs.* 2	1 *vs.* 3	2 *vs.* 3
N	26	44	36	38						
Duration of diabetes (years)	na	19 (4–28)	3 (1–8)	8 (2–24.25)	n.a.	n.a.	n.a.	0.0009 (*)	n.s.	n.s.
HbA_1c_ (mmol/mol)/ (%)	32.5 (31–35)/9.2 (9.1–9.4)	63 (55–75)/8 (7.2–9)	53 (49–65)/7 (6.6–8.1)	54 (44–63)/7.1 (6.2–7.9)	<0.0001 (*)	<0.0001 (*)	<0.0001 (*)	0.0044 (n.s.)	0.0014 (n.s.)	n.s.
SBP (mmHg)	121 (113–128)	125 (119.5–130)	135 (130–149.5)	120.5 (113.5–129)	n.s.	<0.0001 (*)	n.s.	0.0003 (*)	n.s.	0.0001 (*)
DBP (mmHg)	73 (69–79)	76.5 (69–80)	80 (74–85)	71 (66.5–77)	n.s.	0.0156 (n.s.)	n.s.	0.0441 (n.s.)	n.s.	0.0002 (*)
T. Cholesterol (mmol/L)	4.8 (4.13–5.5)	4.25 (3.8–4.7)	4 (3.4–4.68)	4.35 (3.7–5.1)	0.0465 (n.s.)	0.0079 (n.s.)	n.s.	n.s.	n.s.	n.s.
Creatinine (mmol/L)	79 (71.25–95)	70 (65.5–83)	76 (70–85.75)	68 (58–76.25)	0.0282 (n.s.)	n.s.	0.0014 (n.s.)	n.s.	n.s.	0.0136 (n.s.)
ACR (g/mol)	0 (0–0.8)	0.5 (0–1.33)	0.9 (0.5–2.7)	0.6 (0.4–0.96)	n.s.	0.0001 (*)	0.0130 (n.s.)	0.0101 (n.s.)	n.s.	0.0403 (n.s.)
GFR (mL/min/1.73 m^2^)	74 (70–83)	89 (78–113)	85.5 (68–103)	88.5 (75–112)	0.0005 (*)	n.s.	0.0024 (n.s.)	n.s.	n.s.	n.s.
Fasting C-Peptide (pmol/L)	367 (236.5–581)	<66	924 (775–1295)	533 (365–681)	n.a.	0.0009 (*)	n.s.	<0.0001 (*)	n.a.	0.0001 (*)

### 2.6. Reverse Transcription and Quantitative Real-Time-PCR of Mature miRNA

Reverse transcription (RT) reactions are performed using the TaqMan miRNA Reverse Transcription Kit (Life Technologies, Carlsbad, CA, USA) and mature miRNA-specific stem-loop primers [hsa-miR-224, AssayID_002099 (MI0000301), hsa-miR-103, AssayID_000439 (MI0000109)] according to the manufacturer’s instructions. Because the concentration of total RNA in urine has been below the limit of accurate quantitation by spectrophotometry, a fixed volume of 3 µL total RNA was used for cDNA preparation, as previously described [34]. For generation of standard curves, synthetic single-stranded RNA oligonucleotides corresponding to the mature miRNA sequences were purchased from Sigma-Aldrich. A serial of 10-fold dilutions of each oligonucleotide were made in water and run in parallel with the urine samples.

Quantitative real-time PCR (qPCR) was performed in 96-well plates by use of the AIB 7500 instrument (Applied Biosystems, Foster City, CA, USA). The qPCR reaction mixture (20 µL reaction volume) includes 1.33 µL product from RT reaction, 1 µL TaqMan MicroRNA Assay (Life Technologies, Carlsbad, CA, USA), 10 µL of TaqMan 2× Universal PCR Master Mix (no AmpErase) (Life Technologies, Carlsbad, CA, USA) and 7.67 µL nuclease-free water. Amplification was performed under following conditions: 95 °C for 10 min, followed by 40 cycles of 95 °C for 15 s and 60 °C for 1 min. The cycle threshold (*C*_t_) values were calculated with the SDS 2.1 software (Applied Biosystems, Foster City, CA, USA), with the automatic setting for automatic assigning of baseline and threshold for *C*_t_ determination. Each patient sample, sample with spike-in *cel-miR-39* and dilutions of synthetic miRNA for generation of standard curve were run in triplicates. *C*_t_ measurements with high standard deviation between triplicates were considered outliers and excluded from further analysis.

### 2.7. Absolute Quantification of miRNA and Data Normalization against Spiked-in Synthetic C. elegans miRNA Control

Standard curves for each miRNA of interest were generated by plotting *C*_t_ values of the dilutions of synthetic miRNA *vs.* number of copies per reaction followed by exponential fitting. For normalisation of thus quantified miR-103 and miR-224 levels, a median normalization procedure with spike-in *cel-miR-39* was performed as previously described [34]. In brief, a normalisation factor for each sample was defined as Δ*C*_t_ of mean *cel-miR-39* run in triplicate to the median of all *cel-miR-39* that were run in parallel. Quantifications of miRNA in patient samples were then normalised by dividing by 2^Δ*C*t^.

### 2.8. Statistical Analysis

Statistical analyses were performed using MatLab and its statistical toolbox (The MathWorks, Inc., Natick, MA, USA). The individual miRNA copy number was calculated based on 1.7 µL of urine. Areas under the curves (AUCs) for insulin, glucose and C-peptide were calculated using the trapezoidal rule. Data are given as median and interquartile range (IQR) and were compared by Mann-Whitney *U* test and Spearman correlation analysis. Repeated samples of urine were analyzed using Wilcoxon signed rank test for paired data. Categorical data such as medication usage was compared using Fisher’s exact test. The potential of miRNA to discriminate between normal controls and participants of the T1DM, T2DM or HNF1A-MODY mutation carrier groups or a pooled group thereof were assessed by receiver operating characteristic (ROC) analysis. In the ROC analysis, each level of miRNA is tested as a potential cut-off to distinguish between two groups. The resulting sensitivity and specificity is presented as a curve where an AUC = 1 would indicate perfect discrimination. An association between miRNA and renal indices was investigated by linear regression analysis and Spearman correlation. Hypotheses tests were considered statistically significant if *p* < 0.05.

## 3. Results and Discussion

### 3.1. Clinical Characteristics of All Groups

A total of 144 individuals participated in the study. The clinical characteristics of the HNF1A-MODY mutation carriers (*n* = 38), T1DM (*n* = 44), T2DM (*n* = 36) and controls (*n* = 26) are contained in Table 1. The HNF1A-MODY carriers, T1DM and control groups were matched for age [41.5 years (21–52), 37 years (23–49.5) and 38 years (30–44), respectively, *p* = n.s.] and BMI [24.1 kg/m^2^ (21.8–25), 25 kg/m^2^ (22–27) and 23.4 kg/m^2^ (22–27), respectively, *p* = n.s.].

In terms of metabolic parameters, the HNF1A-MODY carriers, T1DM and control groups were comparable for systolic blood pressure (SBP), diastolic blood pressure (DBP), high density lipoprotein (HDL) cholesterol, total cholesterol, low density lipoprotein (LDL) cholesterol and triglyceride level. As expected, the T2DM group had a higher DBP [median [IQR] 80 mmHg (73–85)) and triglyceride level (1.24 mmol/L (0.8–2.68)] than all the remaining groups (*p* < 0.05). There was a significantly lower use of secondary preventative medication amongst both the HNF1A-MODYmutation carriers and T1DM group when compared to the T2DM group. Specifically, in the HNF1A-MODY mutation carriers there was lower use of aspirin (31.5% *vs.* 69.4%, *p* ≤ 0.05), anti-hypertensive agents (15.7% *vs.*58.3% *p* ≤ 0.05) and lipid lowering medications in particular statin therapy (69.4% *vs.* 23.6%, *p* ≤ 0.05) when compared to the T2DM group. Likewise, in the T1DM group there was a lower use of aspirin (36.3% *vs.* 69.4%, *p* ≤ 0.05) and anti-hypertensive agents (34% *vs.* 58.3% *p* ≤ 0.05) when compared to the T2DM group.

As expected, the T1DM group had the highest HbA_1c_ when compared to all remaining groups [63 mmol/mol (8%) (55–75) / (7.2–9); *p* < 0.05] with an undetectable C-peptide level (<66 mmol/L). The T2DM group had a lower OGIS level than the HNF1A-MODY mutation carriers [295 mL/min/m^2^ (223–344) *vs.* 355 mL/min/m^2^ (296–410) *p* < 0.05] in keeping with the presence of insulin resistance.

### 3.2. miR-224 is Detectable in Urine and is Highly Expressed in HNF1A-MODY Mutation Carriers and Participants with T1DM

**Figure 1:**
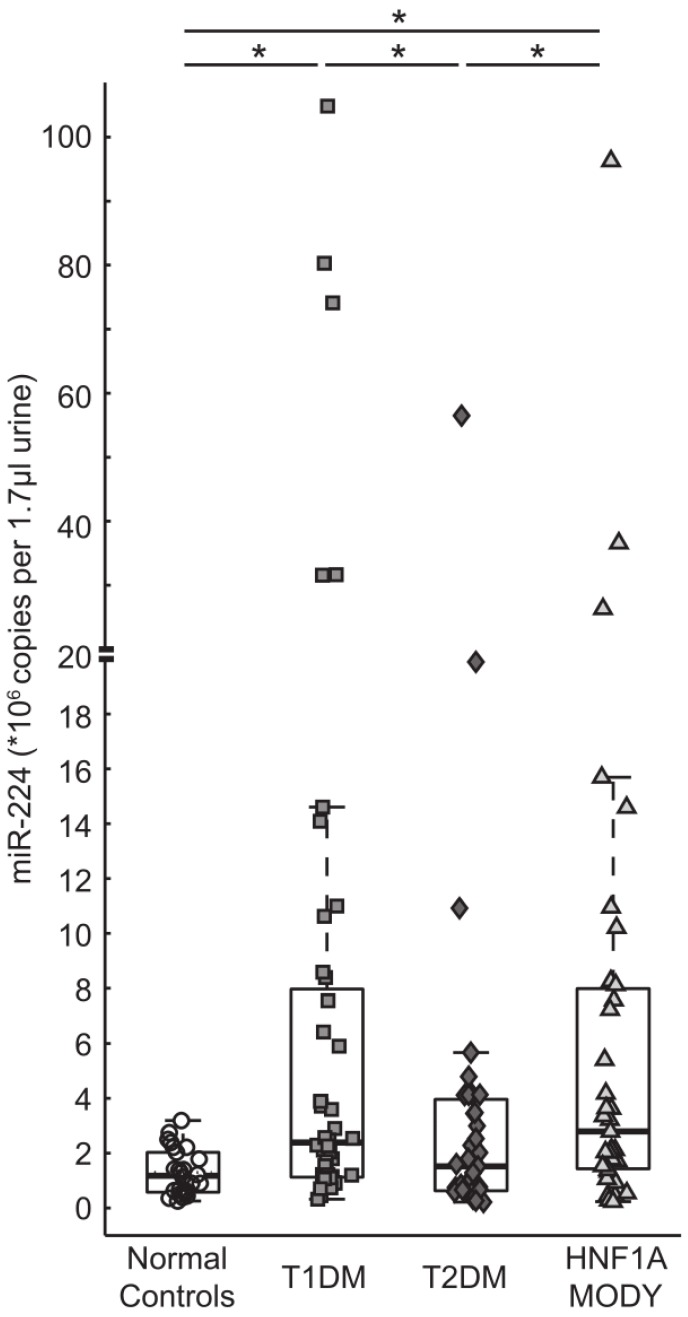
miR-224 levels detected in urine. Copies of miRNA per reaction were determined by quantitative qPCR in urine samples of T1DM (*n* = 44), T2DM (*n* = 36), HNF1A-MODY (*n* = 38) and normal control (*n* = 26) subjects. Box plots depict median and inter quartile range. The HNF1A-MODY mutation carriers had the highest median value of miR-224 and was significantly different from normal controls showing the lowest median value. In addition, the T1DM group had a higher median value than normal controls. miR-224 expression levels in the T2DM group were significantly lower than in T1DM group and the HNF1A-MODY mutation carriers but were similar to normal controls. (* significant differences by Mann-Whitney test).

In our initial report, we identified a strong association between elevated serum levels of miR-224 and the presence of a HNF1A-MODY mutation [25]. To test whether miR-224 was also detectable in the urine of participants with diabetes, RNA from human urine samples was isolated and hsa-miR-224 expression was quantitated via TaqMan qPCR assays. Samples were measured in triplicate using synthetic miR-224 single-stranded RNA Oligonucleotides as standards to obtain absolute miRNA copy numbers that were furthermore normalized against spiked-in synthetic *C. elegans* miRNA control. miR-224 levels were detected at a significantly higher level in the urine of HNF1A-MODY mutation carriers when compared to the control group [2790 × 10^3^ copies per 1.7 µL (1430–7990 × 10^3^) *vs.* 1180 × 10^3^ copies per 1.7 µL (575–2030 × 10^3^), *p* < 0.05] (Figure 1). In a subgroup of patients, a repeat urine sample was attained during clinical follow up (median follow up; 16 months [4–36 months]) and analyzed to determine repeat levels of miR-224. On repeat analysis, there was no significant difference in expression levels for miR-224 [(initial sample) 660 × 10^3^ copies per 1.7 µL *vs.* (subsequent sample) 710 × 10^3^ copies per 1.7 µL, *p* = 0.5], again emphasizing the robust nature of miR-224. In addition, we compared HNF1A-MODY mutation carriers with the highest levels of miR-224 to those having a “low to normal” level for all clinical parameters including age, duration, BMI and HbA_1c_. Findings demonstrate that participants with the highest miR-224 values have measurements within the same range as the “low to normal” group for the clinical parameters studied.

We also determined urine levels of miR-224 in individuals with T1DM and T2DM (Figure 1). miR-224 was significantly elevated in the urine of individuals with T1DM when compared to controls [2380 × 10^3^ copies per 1.7 µL (1120–7970 × 10^3^) *vs.* 1180 × 10^3^ copies per 1.7 µL (575–2030 × 10^3^), *p* < 0.05], but there was no significant difference noted in miR-224 levels in the T1DM population studied when compared to the HNF1A-MODY group [2380 × 10^3^ copies per 1.7 µL (1120–7970 × 10^3^) *vs.* 2790 × 10^3^ copies per 1.7 µL (1430–7990 × 10^3^), *p* = 0.8]. miR-224 expression levels were significantly higher in both the HNF1A-MODY mutation carriers and T1DM groups when compared to the T2DM group [HNF1A-MODY mutation carriers: 2790 × 10^3^ copies per 1.7 µL (1430–7990 × 10^3^) *vs.* 1520 × 10^3^ copies per 1.7 µL (630–3960 × 10^3^), *p* < 0.05] and [T1DM: 2380 × 10^3^ copies per 1.7 µL (1120–7970 × 10^3^) *vs.* 1520 × 10^3^ copies per 1.7 µL (630–3960 × 10^3^), *p* < 0.05].

**Figure 2:**
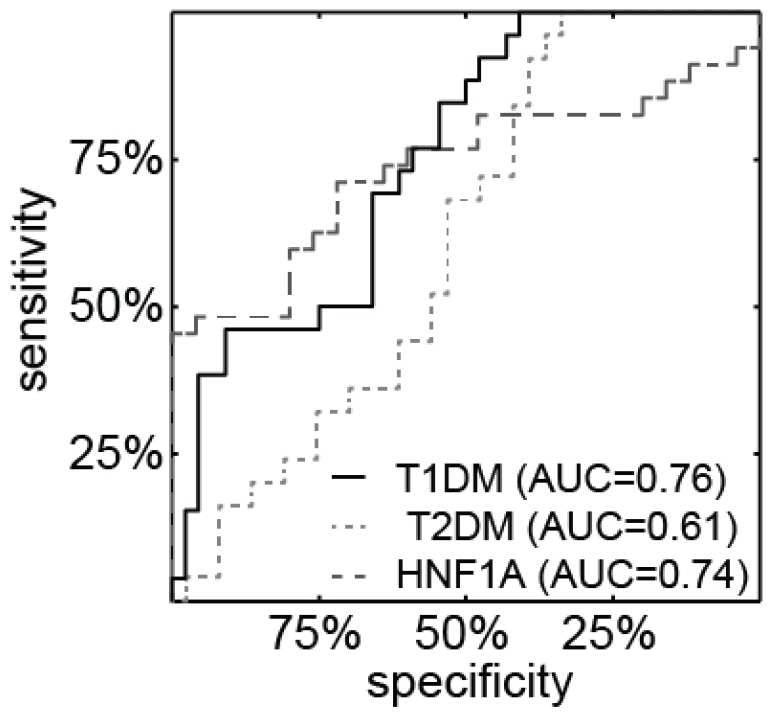
ROC analysis of miR-224. Levels of miR-224 in urine differentiated the normal control group from the T1DM, HNF1A-MODY mutation carrier and T2DM groups, respectively.

The ROC analysis for miR-224 in theHNF1A-MODY mutation carriers, T1DM, T2DM and normal control groups are presented in Figure 2. miR-224 can reasonably differentiate HNF1A-MODY from normal controls (AUC = 0.74) as well as T1DM from normal controls (AUC = 0.76). Furthermore, ROC analysis of pooled groups with diabetes (T1DM, T2DM and HNF1A-MODY mutation carriers) indicates that miR-224 can distinguish between normal controls and diseased patients (AUC = 0.72).

### 3.3. miR-103 is Detectable in Urine and is Highly Expressed in Patients with Diabetes

**Figure 3:**
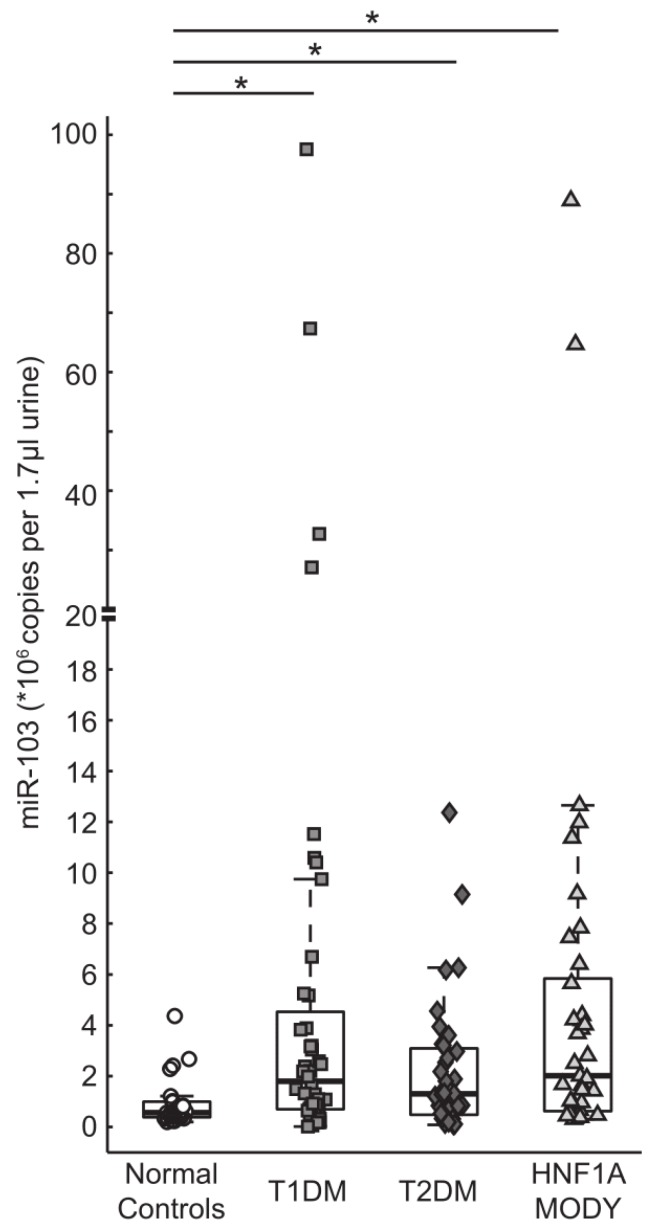
miR-103 levels detected in urine. Copies of miRNA per reaction were determined by quantitative qPCR in urine samples of T1DM (*n* = 44), T2DM (*n* = 36), HNF1A-MODY mutation carriers (*n* = 38) and normal control (*n* = 26) subjects. Box plots depict median and inter quartile range. Levels of miR-103 were statistically higher in all disease groups compared to normal controls where HNF1A-MODY mutation carriers showed the highest expression values (* significant differences by Mann-Whitney test).

We also found strongly elevated levels of miR-103 in the urine of HNF1A-MODY mutation carriers when compared to the control group [median [IQR] 2020 × 10^3^ copies per 1.7 µL (622–5840 × 10^3^) *vs.* 559 × 10^3^ copies per 1.7 µL (385–997 × 10^3^) *p* < 0.05] (Figure 3). Similar to miR-224, we found no significant difference on subsequent measurement for miR-103 [(initial sample) 390 × 10^3^ copies per 1.7 µL *vs.* (subsequent sample) 260 × 10^3^ copies per 1.7 µL, *p* = 0.6].

We next determined urine levels of miR-103 in individuals with T1DM and T2DM. There were significantly higher levels of miR-103 in the urine of individuals with T1DM and T2DM when compared to controls [T1DM: 1800 × 10^3^ copies per 1.7 µL (698–453 × 10^3^) *vs.* 559 × 10^3^ copies per 1.7 µL (385–997 × 10^3^), *p* < 0.05) and [T2DM: 1300 × 10^3^ copies per 1.7 µL (478–309 × 10^3^) *vs.* 559 copies per 1.7 µL (385–997 × 10^3^), *p* < 0.05], respectively. There was no difference noted in miR-103 expression levels between all diabetes cohorts studied (T1DM, T2DM and HNF1A-MODY mutation carriers) (Figure 3).

### 3.4. Correlation of Urine miR-224 and miR-103 Levels with Clinical and Biochemical Characteristics

**Figure 4:**
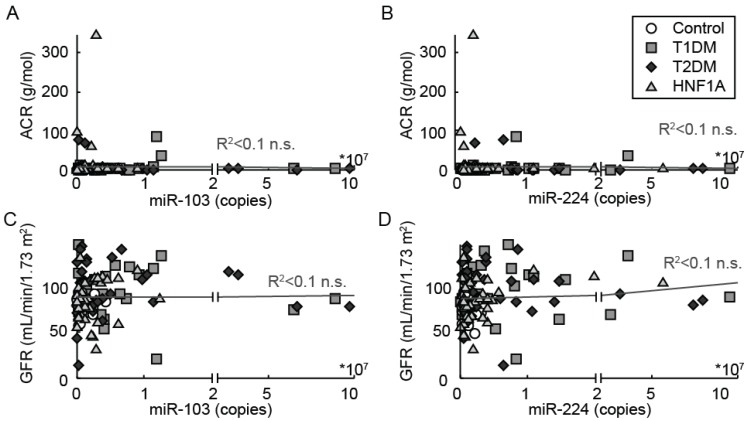
Correlation of miR-224 and miR-103 with renal indices; eGFR/ACR. Linear regression analysis indicates no statistically significant association of miR-224/-103 with GFR/ACR in the HNF1A-MODY mutation carrier, T1DM, T2DM and normal control groups; n.s. not significant.

**Figure 5:**
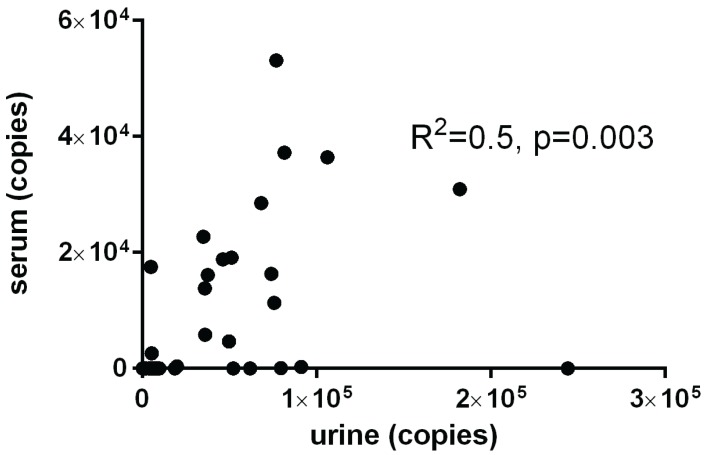
Correlation of miR-224 in the serum and urine of a matched cohort of HNF1A-MODY mutation carriers (*n* = 30) and normal controls (*n* = 8). Spearman correlation analyses demonstrated a significant correlation between serum and urine for miR-224 (*R*^2^ = 0.5, *p* < 0.003).

We next performed Spearman correlation analyses to identify any correlation of urine miRNA levels with clinical and biochemical parameters (Supplementary Table S2). Since any published work available to date on urinary miRNA has been performed in the setting of renal pathology, we analyzed any potential correlation of miR-224 and miR-103 levels with indices of renal disease. The median eGFR was >60 mL/min/1.73 m^2^ in all groups signifying the absence of overt renal disease. Likewise, the median ACR values in all patients studied did not reveal microalbuminuria or overt albuminuria. The miR-224 and miR-103 levels in HNF1A-MODY mutation carriers, T1DM or T2DM did not correlate with the renal indices; ACR or eGFR (Figure 4). In addition, there was no correlation detected between miR-224 and miR-103 and urinary osmolality (miR-224; ρ = 0.04, *p* = 0.6 and miR-103; ρ = −0.002, *p* = 0.9). Serum and urine miRNA analysis performed in matched cohort samples, using identical miRNA quantification methods revealed a significant correlation between serum and urine miRNA levels (miR-224: ρ = 0.55, *p* ≤ 0.01 and miR-103: ρ = 0.38, *p* = 0.01). Figure 5 demonstrates the statistically significant association between serum and urine for miR-224 in a cohort of HNF1A-MODY mutation carriers and normal controls.

## 4. Discussion

miRNA represent an important mechanism in the regulation of gene expression and the potential to manipulate this expression has significant implications in the management of disease states including diabetes. In this study, we report, for the first time, detection of circulating diabetes-associated miR-224 and miR-103 in the urine of patients, notably in the absence of renal pathology. This is also the first study to detect miRNA in the urine of a large cohort of HNF1A-MODY mutation carriers. HNF1A-MODY is a monogenic form of diabetes and therefore an ideal model for the study of the beta-cell. HNF1A-MODY is primarily a disorder resulting in beta-cell dysfunction, with mutation carriers lacking features of the metabolic syndrome such as insulin resistance, as opposed to the multifactorial aetiology synonymous with T2DM. We chose to study miR-224 and miR-103 in particular based on preliminary work performed in our laboratory whereby the endogenous expression of HNF1A in INS-1 cell lines resulted in the elevated expression of both miR-224 and miR-103 [25].

Of interest, we have found that miR-224 is highly expressed in the urine of patients with T1DM and HNF1A-MODY mutation carriers when compared to both the T2DM and the normal control cohorts. miR-224 is a novel miRNA in the field of diabetes. To date, it has been shown that miR-224 is aberrantly expressed in a wide variety of malignancies including prostatic, hepatocellular, renal cell and colorectal. It is known to promote cell migration, proliferation, and invasion and has been shown to target TGF-β signaling via the Smad 4 pathway [35,36,37,38]. The elevated expression of miR-224 in both HNF1A-MODY mutation carriers and T1DM is not surprising given that both share certain traits. Beta-cell failure and ultimately a reduction in beta-cell mass are noted in both HNF1A-MODY mutation carriers and T1DM [39,40]. In contrast to T2DM, which is a *relative* insulin deficient state with the pathology attributed largely to insulin resistance, it is a defect in insulin secretion that is the principal pathology of both HNF1A-MODY mutation carriers and T1DM [41]. This defect predates the clinical manifestation of diabetes as demonstrated by research in the carriers of HNF1A-MODY who were normoglycaemic and likewise in islet antibody positive relatives of those with T1DM [42,43]. We can hypothesize that the altered miR-224 expression profile may be a contributor to the beta-cell dysfunction noted in both HNF1A-MODY mutation carriers and T1DM. We can speculate that miR-224, as a marker of beta-cell demise, has the potential to be an important clinical development given the current inability to perform pancreatic biopsies in humans and lack of beta-cell imaging techniques. There are currently no available biomarkers of beta-cell demise.

miR-224 was significantly elevated in the urine of HNF1A-MODY mutation carriers when compared to the T2DM cohort studied. We therefore propose that the clinical utility of miR-224 may be further expanded as an additional screening tool to decipher who should be screened for HNF1A-MODY genetic testing. This is of clinical relevance, given that it is not feasible to perform genetic testing on all potential mutation carriers, and the greatest clinical challenge is in the discrimination of potential HNF1A-MODY mutation carriers from lean patients with T2DM.

In the current study, miR-103 was highly expressed in the urine of all participants with diabetes when compared to the normal control cohort. To our knowledge, this is the first description of miR-103 in the urine of patients with diabetes. A recently published meta-analysis ranked miR-103 as one of the most common, consistently deregulated miRNA in diabetes across multiple tissue types including pancreatic, adipose and serum [44]. Our novel finding of such a well-established diabetes-associated marker in urine of patients with both mono- and polygenic forms of diabetes lends credence to the validity of the methodology employed to detect miRNA in the current study. Whilst the pathway regulated by miR-224 is not fully elucidated, the principal targets of miR-103 have been investigated extensively. Caveolin 1 is a known target of miR-103; *in vitro* studies have previously demonstrated that caveolin 1 interacts with the insulin receptor improving insulin mediated phosphorylation of IRS-1 [5]. Cav-1 deficient mice develop post-prandial hyperglycaemia and insulin resistance. It may be that the increased miR-103 expression in diabetes is an attempt to surmount the lack of insulin with enhanced insulin signaling.

In the current study, we can only speculate as to the origin of miR-224 and miR-103 in the urine of individuals with diabetes. The miRNA detected in the urine may have originated from beta-cells or indeed from tissues affected by diabetes such as liver, muscle, or kidneys. It is also possible that miRNA are actively secreted by the kidneys. A likely explanation is that miRNA derive in urine (at lower quantities than in serum) from the blood through the process of glomerular filtration. The approximate molecular weight of miRNA is 6.2–7.2 kDa [45]. The threshold of the glomerular filtration barrier is approximately 60 kDa. Therefore, a substantial portion of circulating miR-224 and miR-103 could be ultra-filtrated through the kidneys.

The utilization of urine as a biofluid has multiple advantages in the clinical setting. Urine sampling is non-invasive and it is collected at every diabetes and nephrology clinic. Urine is not subject to haemolysis. This of utmost importance, as blood contamination and haemolysis are the two most common artefacts encountered in miRNA detection in fluids acquired by invasive means (serum/plasma/CSF) [20,45,46]. A publication by Mall *et al.* showed that urinary miRNA remain stable under harsh conditions including freeze/thaw cycles and prolonged storage at room temperature. Although there may be some degradation over time, lower levels have been shown to remain detectable [30]. We have performed repeat urinary miR-224 and miR-103 measurements on a subset of participants. The repeat samples were drawn on separate days in a non-fasting state. There was no significant difference noted between both measurements. These particular urinary miRNA appear to be robust and independent of dietary restriction which is important in a clinical setting.

A limitation of the current investigation is that we cannot provide a definitive causation for the differential expression of urinary miR-224 between the different forms of diabetes. However, reassuringly, we have demonstrated that the elevated expression of miR-224 and miR-103 levels is a common phenotype *in vitro*, in serum and now in the urine of HNF1A-MODY mutation carriers. As aforementioned, HNF1A-MODY represents an ideal beta-cell model. The establishment of gene manipulated *in vitro* and rodent models may facilitate the functional analyses of such miRNA and promote further translational research for the diagnosis and treatment of diabetes. A further strength of this study is the inclusion of both mono- and polygenic diabetes forms.

## 5. Conclusions

In conclusion, we here provide the first proof-of-concept study that miRNA can be readily detected in the urine of participants with diabetes. miR-224 is a novel diabetes-associated miRNA which is highly expressed in the urine of HNF1A-MODY mutation carriers and a T1DM cohort. We surmise that the differential expression levels of miR-224 in both insulin deficient states may be an attempt to compensate for beta-cell demise. A further novel finding of the current study is the detection of the well-established diabetes-associated miR-103 in urine. Finally, our study highlights the benefits in the use of urine as a biofluid for miRNA detection in the clinical setting.

## Supplementary Files

Supplementary File 1
