# Supplementary material for: MicroRNA-224 is Readily Detectable in Urine of Individuals with Diabetes Mellitus and is a Potential Indicator of Beta-Cell Demise
Source: Genes (Basel). 2015 Jun 23;6(2):399–416. doi: 10.3390/genes6020399 (PMC4488671; doi:10.3390/genes6020399)
Supplement: Supplementary File 1 [file genes-06-00399-s001.pdf]

Supplement Materials

Table S1. The Ct range values for the qPCR in urine.

	Normal controls	T1DM	T2DM	HNF1A-MODY
Ct range (qPCR) miR-224 (delta)	29.4–33.7(4.34)	26.5–33.6 (7.1)	26.4–34.2(7.8)	25.9–33.1(7.3)
Ct range (qPCR) miR-103(delta)	32.2–36.4 (4.2)	25.2–36.7 (11.6)	30.1–37.4 (7.3)	28.1–35.8 (7.7)

Table S2. Correlation of urine miR-224 and miR-103 levels with clinical and biochemical characteristics.

	miR-103 in urine								miR-224 in urine							
	Normal controls		T1DM		T2DM		HNF1A-MODY		Normal controls		T1DM		T2DM		HNF1A-MODY	
	rho	*p*	rho	*p*	rho	*p*	rho	*p*	rho	*p*	rho	*p*	rho	*p*	rho	*p*
miR-224 in urine (copies per reaction)	0.14	0.49	0.41	* <0.01	0.26	0.13	0.57	* <0.01	NA	NA	NA	NA	NA	NA	NA	NA
Age (yrs.)	0.39	0.05	−0.10	0.53	−0.46	* <0.01	0.10	0.57	0.19	0.36	−0.13	0.41	−0.20	0.25	−0.14	0.44
Duration (yrs.)	NA	NA	−0.10	0.53	−0.13	0.45	−0.12	0.47	NA	NA	−0.22	0.15	−0.04	0.80	−0.36	* 0.04
BMI (kg/m^2)	−0.07	0.74	0.04	0.81	0.07	0.69	0.06	0.73	0.09	0.66	0.28	0.07	−0.19	0.28	−0.13	0.47
HbA_{1c} (mmol/mol)/%	0.25	0.24	0.12	0.43	0.27	0.11	−0.29	0.10	0.29	0.15	0.22	0.15	−0.10	0.58	−0.12	0.52
SBP (mmHg)	0.05	0.80	−0.32	* 0.03	0.12	0.49	0.02	0.90	0.41	* 0.04	−0.11	0.49	−0.32	0.06	0.18	0.32
LDL (mmol/L)	0.03	0.90	−0.08	0.58	−0.31	0.07	−0.08	0.68	0.50	* 0.02	−0.10	0.53	−0.19	0.28	−0.11	0.56
ACR (g/mol)	0.38	0.06	−0.14	0.41	0.13	0.46	−0.03	0.88	−0.09	0.66	−0.19	0.26	−0.04	0.80	0.01	0.94
GFR (ml/min/1.73m^2)	−0.16	0.49	0.23	0.15	0.18	0.30	0.12	0.50	−0.25	0.26	0.00	1.00	0.26	0.13	0.20	0.27
Insulin (pmol/L)	−0.47	* 0.02	0.00	0.99	0.18	0.31	0.03	0.85	0.03	0.88	−0.15	0.35	0.19	0.29	0.04	0.83
Glucose (mmol/L)	0.02	0.94	0.27	0.10	0.13	0.47	−0.29	0.10	0.13	0.53	0.13	0.41	−0.23	0.19	−0.09	0.63
